# Enhanced Kat3A/Catenin transcription: a common mechanism of therapeutic resistance

**DOI:** 10.20517/cdr.2019.32

**Published:** 2019-09-19

**Authors:** Andrea Bild, Jia-Ling Teo, Michael Kahn

**Affiliations:** 1 Department of Medical Oncology & Therapeutics Research, Beckman Research Institute of the City of Hope, Duarte, CA 91010, USA.; 2 Department of Molecular Medicine, Beckman Research Institute of the City of Hope, Duarte, CA 91010, USA.

**Keywords:** Kat3 coactivator, CREB-binding protein, p300, therapy resistance, stem cell, cancer stem cell

## Abstract

Cancers are heterogeneous at the cellular level. Cancer stem cells/tumor initiating cells (CSC/TIC) both initiate tumorigenesis and are responsible for therapeutic resistance and disease relapse. Elimination of CSC/TIC should therefore be able to reverse therapy resistance. In principle, this could be accomplished by either targeting cancer stem cell surface markers or “stemness” pathways. Although the successful therapeutic elimination of “cancer stemness” is a critical goal, it is complex in that it should be achieved without depletion of or increases in somatic mutations in normal tissue stem cell populations. In this perspective, we will discuss the prospects for this goal via pharmacologically targeting differential Kat3 coactivator/Catenin usage, a fundamental transcriptional control mechanism in stem cell biology.

## INTRODUCTION

Cancer is a major contributor to worldwide mortality^[1]^. There are minimally four broad resistance-inducing strategies that are employed by cancer cells including: (1) direct target reactivation; (2) activation of signals upstream or downstream of oncogenes; (3) engagement of parallel oncogenic pathways; and (4) adaptive survival mechanisms. Despite tremendous advances in targeted therapeutics and personalized medicine, which have significantly increased progression free survival, maximum clinical success as defined by overall survival or “cures”, remain limited due to therapeutic resistance^[2]^. These resistance mechanisms can be attributed to a subpopulation of self-renewing, highly tumorigenic, drug-resistant cancer stem cell/tumor initiating cell (CSC/TIC), in which therapeutic pressure leads to the selection of therapy resistant clone^[3–8]^.

### Stem cells and cancer stem cells

All stem cells by definition, have the capacity to both self-renew (i.e., make at least one identical copy of itself at each division) as well as to differentiate into more mature, albeit less potent, specialized cells. The concept of CSC is not new. Cohnheim, more than 150 years ago, proposed that cancer might arise from rare cells with stem cell-like properties^[9]^. The existence of CSC has now been demonstrated in many tumor types including leukemia, brain, breast, bladder, prostate, colon, etc., where their presence has been associated with disease recurrence, multidrug resistance and metastasis^[10]^. Therefore a critical goal to change the course of cancer therapy is to develop strategies to safely eliminate CSC without deleterious effects to normal spermatogonial stem cell (SSC) populations.

Two mechanisms are proposed to account for the generation of CSC. In the stochastic model, cancer cell plasticity endows non-CSC with the ability to dedifferentiate into CSC. Alternatively, in the hierarchical model, CSC are able to self-renew thereby expanding the CSC pool from which escape mutants can be selected. CSC and SSC share multiple characteristics, including self-renewal and the potential to differentiate. As previously pointed out, the term “cancer stem cell” does not have to refer to the cell of origin^[11]^. Rather the term CSC refers to cells that have “stem-like” properties. CSC can originate from tissue stem cells, transiently amplifying cells or potentially even differentiated cells^[12]^. SSC, due to their longevity and self-renewing properties, have a far greater propensity to accumulate carcinogenic mutations, which could markedly influence the behavior of those cells, e.g., accelerate self-renewal via a switch from asymmetric to symmetric division, which will be further discussed^[13,14]^. It is also possible that the initial mutations occur in SSC, yet the final mutations that confer oncogenesis occur during neoplastic transformation in downstream progeny that have blocks in terminal differentiation^[15]^. Further, interaction with the environment or signaling changes within a cell can lead to epigenetic or phenotypic state changes relevant to CSC generation^[16]^. Regardless of the exact origin of CSC, therapeutic resistance in CSC has been associated with (1) quiescence, as most conventional cytotoxic agents target proliferating cell^[17,18]^; (2) high expression of drug-efflux pumps, e.g., ATP binding cassette (ABC) family transporters^[19]^; (3) increased DNA repair and detoxifying enzymes^[20]^; (4) acquisition of an EMT-like phenotype^[21]^; and (5) utilization of hypoxic niche microenvironments that provide survival fostering signals^[22]^.

Targeting CSC could in principle be accomplished via the targeting of CSC specific cell surface markers or through alternatively “stemness” pathways. Although the successful therapeutic elimination of “cancer stemness” offers enormous promise, it will require significant precision to avoid deleterious effects (e.g., depletion of, or increases in, somatic mutations) in normal SSC populations. Unfortunately in this regard, the similarities between normal adult SSC and CSC far outweigh their differences^[23]^. CSC express similar “stemness” markers and exhibit similar cellular behaviors to SSC as described above. SSC in tissues preferentially inhabit specialized hypoxic niches and are critical for both normal tissue homeostasis and regeneration after injury^[24–26]^. Long-lived SSC are quiescent and rarely become activated under homeostatic conditions, however upon injury to repair damaged tissue, they enter the cell cycle. CSC occupy the same hypoxic niches, thereby competing with normal SSC for this limited environment. The same signal transduction pathways utilized in SSC maintenance, proliferation and differentiation (i.e., Wnt, Notch, Hedeghog, TGFβ/BMP, JAK/Stat, Hippo, FGF/MAPK/PI3K) also regulate CSC^[27–29]^. For both CSC and SSC, there are multiple points of intersection and crosstalk, including feedback and feed forward loops, connecting the various signaling cascades that modulate “stemness” allowing for escape from driver directed therapeutics. These targets and therapies blocking these pathways are summarized in recent reviews^[8,30–32]^.

**Figure 1.**
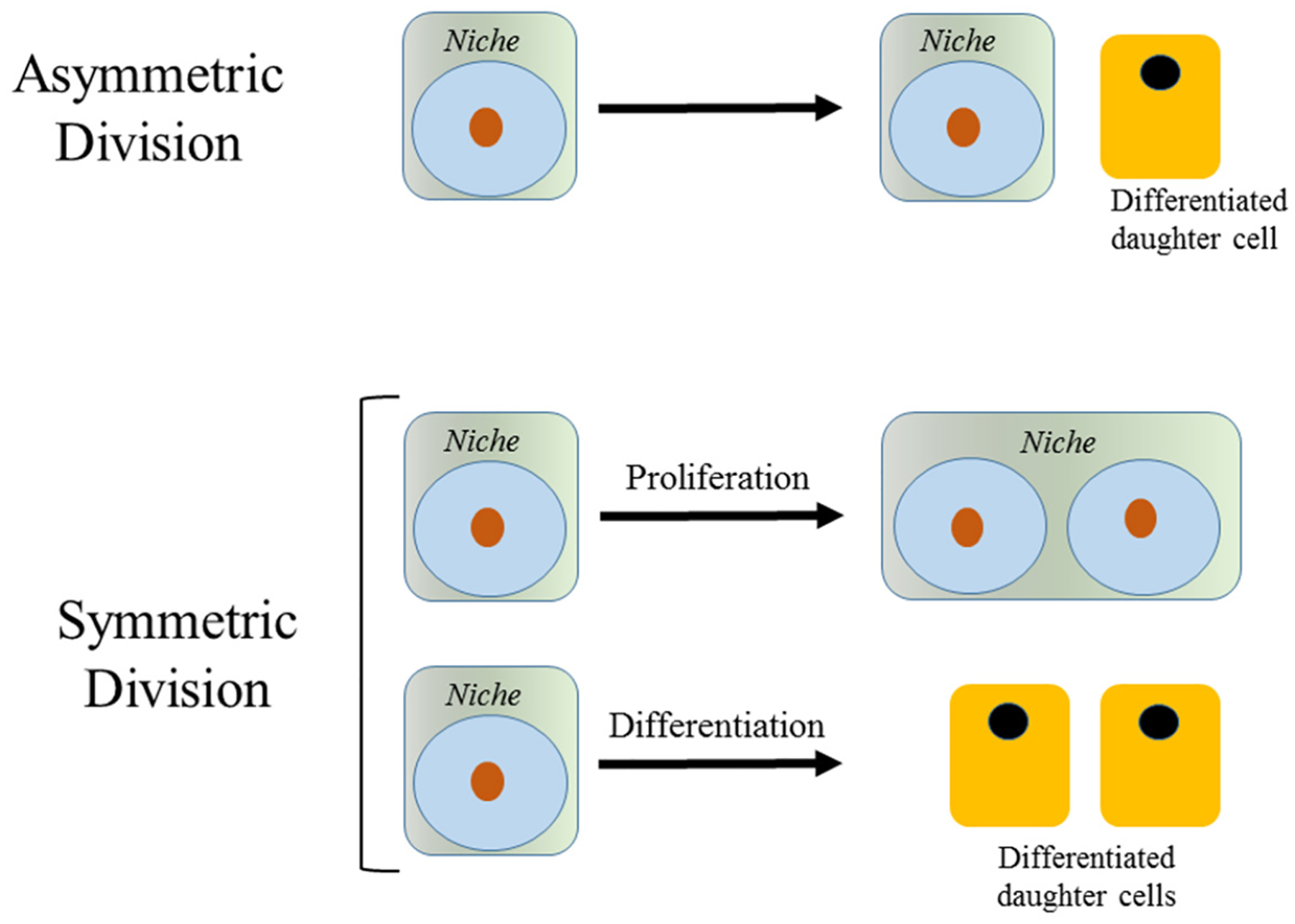
Stem cell divisions. An asymmetric division results in the production of two daughter cells with different cell fates-one a stem cell and the other a diafferentiated daughter cell. There are two modes of symmetric divisions: symmetric non-differentiative divisions generate two daughter cells that remain as stem cells, whereas symmetric differentiative division gives rise to two daughter cells, both of which are differentiated daughter cells

Adult SSC are present in limited numbers. They are believed to be essentially immortal and remain with us for our entire lives. The “dark side” of the immortality of SSC is their capacity to be corrupted into CSC. Like their normal counterpart SSC, CSC exhibit self-renewal capacity and differentiation potential, albeit with aberrant and incomplete differentiation, thereby having the capacity to maintain or renew and propagate a tumor. Under normal homeostatic conditions, long-term SSC divide relatively infrequently, perhaps only once every few months^[33]^ or even less^[34]^. Quiescent SSC, once they enter the cell cycle, can undergo mitosis to give rise to two daughter cells. Mitotic stem cells can divide either symmetrically or asymmetrically [Figure 1]. Ideally, an asymmetric balance is maintained, whereby one of the daughter cells remains in its niche as a stem cell and the other daughter proceeds forward to amplify and subsequently differentiate. However, stem cells (both SSC and CSC) can also undergo symmetric divisions. There are two modes of symmetric division: (1) symmetric non-differentiative divisions, where both daughter cells remain as stem cells in their niche; or (2) symmetric differentiative divisions, where both cells go on to differentiate [Figure 1]. Symmetric division in our essentially “immortal” SSCs, are considered deleterious, leading either to premature exhaustion of the stem cell pool or alternatively increasing the number of DNA lesions accumulated in SSC (via symmetric differentiative and non-differentiative divisions respectively). The preference for long-lived SSC to undergo asymmetric divisions is outlined in the Cairn’s “immortal strand hypothesis”^[35]^, which postulated that the stem cell desires to retain its original uncopied strands of DNA and to pass on the duplicated strands that contain multiple copy errors, inherent in the DNA replication process, to its differentiated daughter cell, thereby minimizing the total number of DNA mutations that accumulate in the long-lived SSC population. In order to make the decision to divide symmetrically versus asymmetrically, a stem cell undergoing mitosis must read an enormous array of information from its environment (e.g., oxygen levels, nutrient levels, circadian cycles, growth factors, adhesion molecules, kinase cascades, cell–cell contacts, etc.). How is all of this information integrated to decide a stem cell’s fate, i.e., to exit quiescence and subsequently divide either asymmetrically or symmetrically, be it a normal SSC or a CSC?

Interestingly, a preference for symmetric over asymmetric divisions appears to be one of the fundamental differences between CSC and SSC. Breast cancer stem cells with p53 mutations preferentially undergo symmetric divisions^[36]^. Loss of the tumor suppressor PTEN leads to premature exhaustion of the normal hematopoietic stem cell population, presumably via increased symmetric differentiative divisions and expansion of the leukemic stem cell population via increased non-differentiative symmetric divisions^[37]^. Indirect perturbation of Notch signaling, via genetic activation of the Hedgehog pathway, also causes an increase in neural stem cell symmetric divisions^[38]^. Symmetric differentiative divisions by “corrupted” SSC prior to the accumulation of additional deleterious mutations generates bona fide CSCand can stochastically eliminate this SSC population. This mechanism prevents non-differentiative symmetric divisions expanding the “pre-CSC” pool. An example of this expansion of the “pre-CSC” pool is represented by clonal hematopoiesis of indeterminate potential (CHIP). CHIP is defined by the presence of somatic hematologic-cancer-associated gene mutations and can be seen in the peripheral blood of at least 10% of people older than 60 years of age without any history of hematologic disorders^[39]^. The presence of CHIP is associated with an increased risk of hematologic cancers and an increased overall mortality^[40]^.

### Wnt/Catenin-dependent transcription and “stemness”

**Figure 2.**
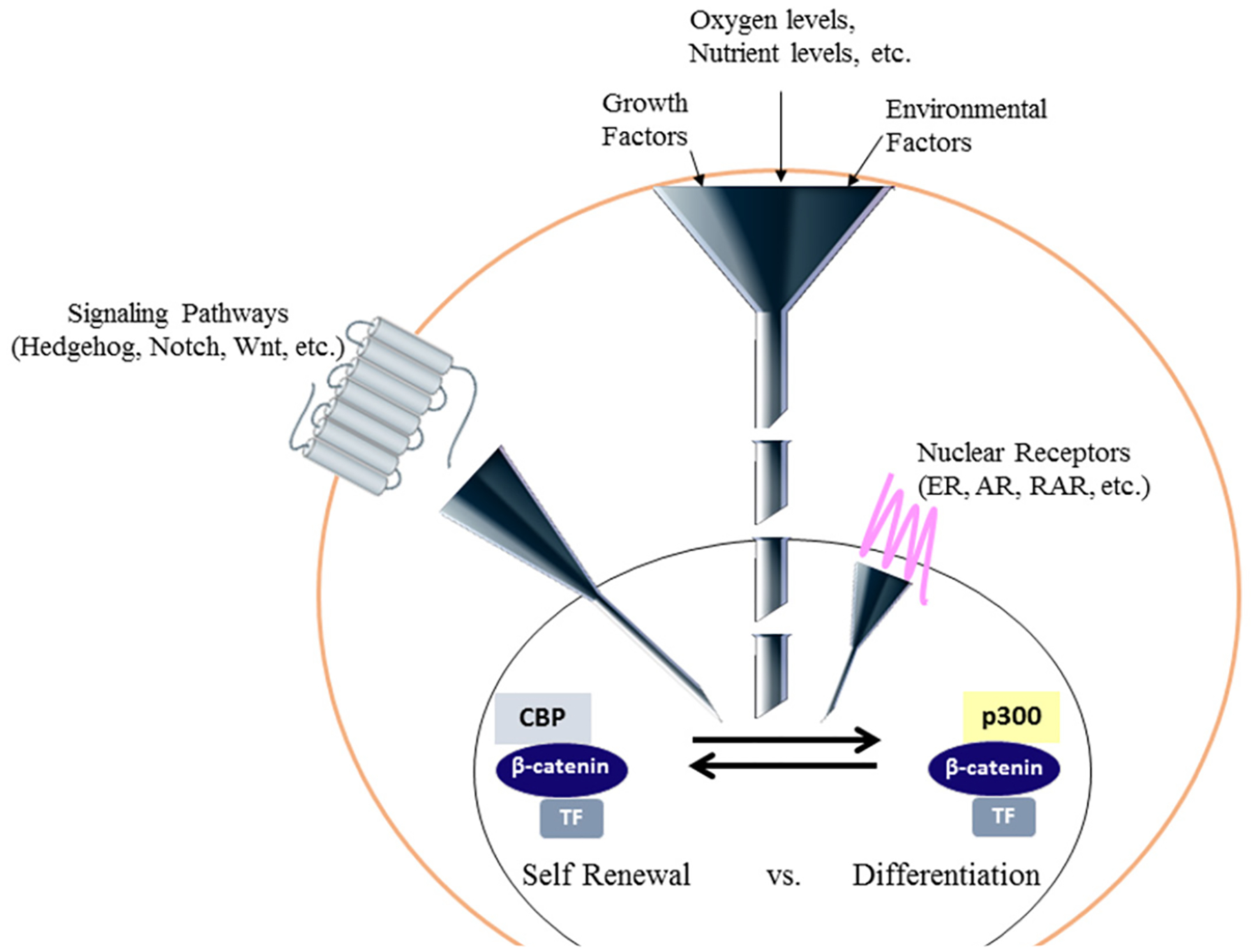
Coordination and integration of multiple signaling cascades is required to regulate the decision of a stem cell to either differentiate or self-renew. Multiple signaling inputs both intrinsic and extrinsic, including nutrient and oxygen levels, growth factors and various signaling cascades must be integrated and funneled down to regulate a transcriptional program either leading to self-renewal or the initiation of differentiation

Wnt signaling is an ancient and highly evolutionarily conserved pathway that is important throughout embryonic development and the life of an organism. It is a very complex signaling cascade^[41]^ that initiates a broad range of intracellular responses broadly classified as either canonical (involving nuclear β-catenin mediated transcription) or non-canonical (planar cell polarity, Ca^2+^/PKC activation)^[42,43]^. Canonical Wnt signaling is generally associated with proliferation and lack of differentiation (for example in cancer), whereas the non-canonical pathway regulates cellular patterning and tissue organization. β-catenin is critical in both pathways via its roles either in the nucleus or cytoskeleton and cytoplasmic membrane, respectively. Although designating Wnt signaling as either canonical or non-canonical allows for simplified conceptual discourse, there is great crosstalk between the two responses, and Wnt crosstalk regulates complex nonlinear networks in development and homeostasis^[44]^. Nuclear β-catenin, although additional catenins, including γ-catenin/plakoglobin, may additionally participate under particular circumstances^[45]^, in transcription is controlled by the so-termed “canonical Wnt” or “Wnt/β-catenin” signaling cascade. Nuclear translocation of β-catenin and its subsequent transcriptional activity can also be induced by non-Wnt signaling. Epithelial to mesenchymal transition, leads to β-catenin nuclear translocation^[46]^, perhaps through down-regulation of β-catenin’s cytoplasmic binding partner E-cadherin^[47]^. Receptor tyrosine kinases^[48]^ and non-receptor tyrosine kinases including Src^[49]^ and Abl^[50]^ can enhance β-catenin-mediated transcription by disrupting the E-cadherin/β-catenin interaction. Prostaglandins^[51]^, hypoxia^[52,53]^, high glucose levels^[54]^, and cholinergic innervation^[55]^ additionally may activate Wnt/β-catenin signaling. A wide range of inputs an influence β-catenin dynamics and β-catenin-dependent transcription^[56–58]^. Balancing self-renewal versus differentiation in SSC, requires signaling from a number of other pathways (e.g., Notch, Hedgehog, JAK/Stat, BMP, Hippo, FGF/MAPK) that must be integrated with nuclear β-catenin signaling [Figure 2]. Wnt signaling is critical in stem cell biology and development^[59]^. However, there is no consensus on whether Wnt signaling is important for either maintenance of potency^[3,60]^ or the differentiation of stem cells^[61]^. Wnt/catenin signaling clearly plays dichotomous roles in SSC biology^[62]^.

### Wnt/Catenin signaling in cancer stem cells and cancer

Wnt signaling plays a critical role in SSC homeostasis^[63]^. Not surprisingly, aberrant regulation of Wnt signaling is a recurrent theme in cancer biology^[64,65]^ and has been implicated in the tumorigenic potential of stem cells.

Continued expression of BIRC5/Survivin, a Wnt target gene, in hES cells is essential for teratoma formation^[66]^. Wnt/β-catenin regulation of telomerase activity endows stem cells and cancer stem cells with unlimited self-renewal capacity^[67]^. Slug, a strong inducer of EMT in tumors, is associated with nuclear accumulation of transcriptionally active β-catenin^[68]^. Over-expression of either of the putative Wnt target gene EMT inducing factors twist and snail increases the expression of CSC markers^[69]^. The connection between enhanced nuclear β-catenin signaling and EMT is strengthened further by the significant number of β-catenin target genes (e.g., S100A4, fibronectin, L1CAM, CD44, MMP7, uPAR, etc.) associated with invasion, migration and metastases^[70]^. Wnt signaling in CSC is associated with metastasis^[71]^, and the regulation of organ specific tropism of CSC during metastasis^[72]^, as well as in the formation of the pre-metastatic niche that nurtures metastasizing CSC^[73]^. Cdx-1^[74]^ and Id2^[75]^, two transcription factors associated with the maintenance of a stem like” state, have been shown to be β-catenin regulated. Many cell surface markers in stem cell and cancer stem cells are direct Wnt targets, including LGR5/GPR49^[76]^, CD44^[77]^, CD24^[78]^, CD133^[79]^, ABC cassette genes^[80,81]^ and EpCAM^[82]^. The first identified CSC in solid tumors had a CD44^high^ CD24^low^ phenotype and comprised a population of breast cancer CSC possessing tumor-initiating capacity^[83]^. These genes and related references are listed in Table 1. Many Wnt signaling related genes are up-regulated in hematopoietic malignancies^[84,85]^ and epigenetic silencing of negative regulators of the Wnt signaling cascade is frequently associated with leukemia^[86]^. Moreover, aberrant activation of tumor associated Wnt/β-catenin signaling has been correlated with resistance to radiation, cytotoxic and targeted chemotherapy^[87,88]^ and most recently checkpoint immunotherapy resistance in multiple tumor types including, melanoma, bladder and head and neck cancers^[89]^. Tumor-intrinsic Wnt/β-catenin signaling mediates cancer immune evasion by preventing T-cell and/or dendritic cell infiltration, migration and function, and thereby resistance to immune checkpoint inhibitors^[90,91]^ and has been shown to maintain T-cells in a differentiated exhausted dysfunctional state^[92]^.

### Targeting Wnt/Catenin signaling in SSC and CSC

Successful pharmacologic manipulation of aberrant catenin-regulated transcription of endogenous “stemness” in SSC and CSC holds enormous potential. However, significant concerns arise in regards to potential deleterious effects on normal SSC populations, including increasing DNA lesions or elimination of normal SSC while attempting to eliminate CSC or activate quiescent or senescent SSC^[23,41,93]^. It may seem obvious to target the Wnt signaling pathway in both SSC and CSC and indeed this has engendered substantial efforts to develop therapeutic agents. Despite these efforts, no therapeutic agents to date specifically targeting the Wnt pathway have been approved for use in patients. A number of factors have thwarted progress in this regard. First, the Wnt signaling cascade is highly complex^[41,42]^. For example, in addition to classical canonical Wnt/β-catenin/TCF transcription, Wnt proteins elicit a variety of alternative non-canonical responses^[94,95]^. Secondly, crosstalk from various non-Wnt factors can also modulate nuclear β-catenin accumulation as previously discussed. Overall, the ability to target Wnt signaling holds enormous potential; however, like the sword of Damocles, it brings substantial risks and concerns as it is also a crucial pathway in normal SSC main tenance and tissue homeostasis.

### Differentiation therapy

All-trans retinoic acid provided a breakthrough differentiation therapy for acute promyelocytic leukemia. However, broad scale success with differentiation therapy has not been achieved to date^[96]^. As stated previously, a preference for symmetric over asymmetric divisions appears to be one of the fundamental differences between CSC and SSC. The question then is: can we safely manipulate endogenous stem cell populations by taking advantage of their preferred modes of division to differentiate away the CSC population without eliminating normal SSCs?

**Figure 3.**
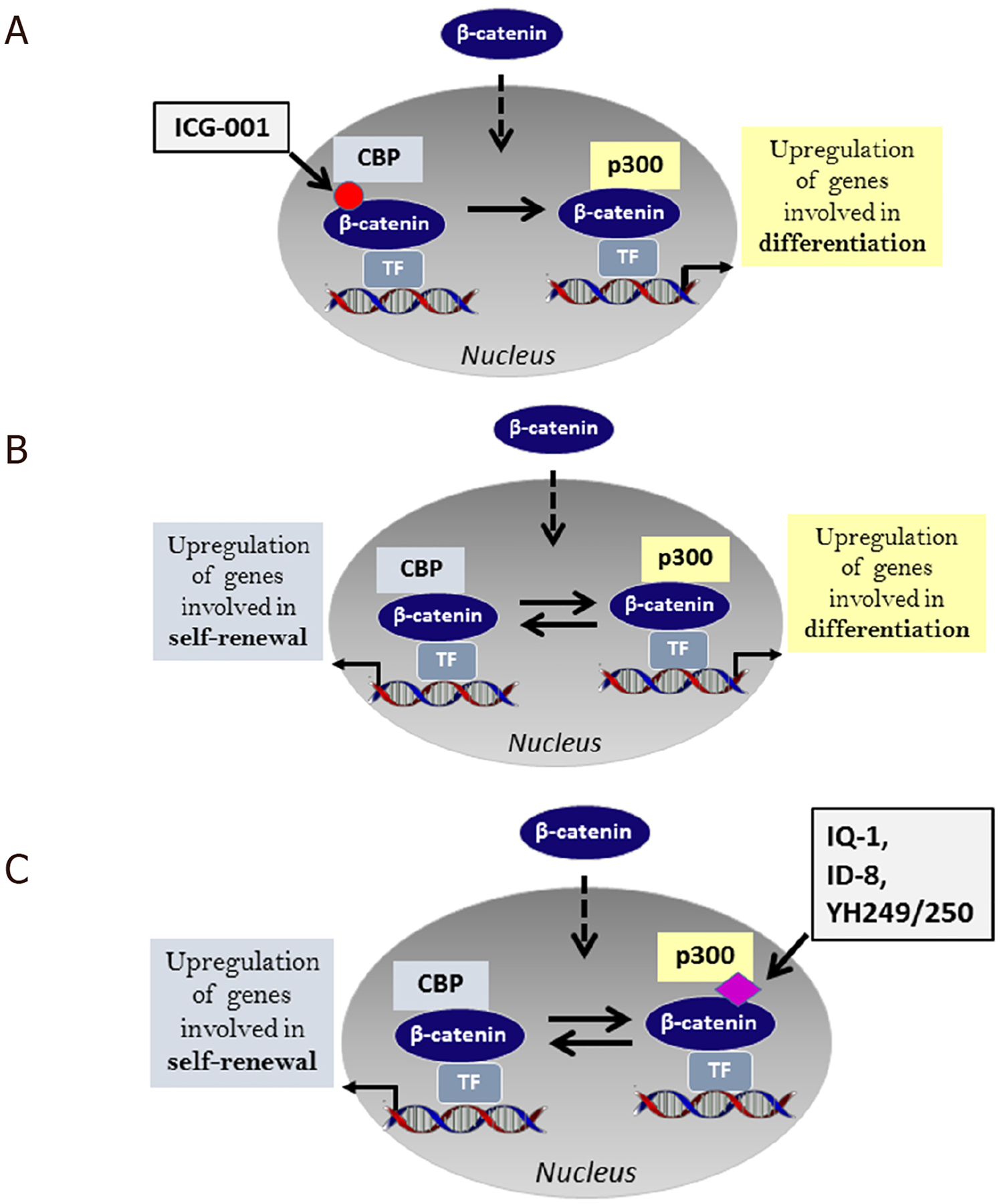
Differential Kat3 coactivator usage. A: ICG-001 specifically disrupts the interaction between CBP and β-catenin. This leads to increased p300/β-catenin transcription, a loss of the capacity to self-renew and the initiation of differentiation; B: β-catenin differential coactivator usage regulates differentiation versus self-renewal. β-catenin usage of either CBP or p300 leads to transcriptional activation of genes that are critical for self-renewal or differentiation respectively; C: IQ-1, ID8 (indirectly), and YH 249/250 (directly) disrupt the p300/β-catenin interaction. Selectively antagonizing the p300/β-catenin interaction enhances CBP/β-catenin transcription thereby favoring self-renewal

In order to form a transcriptionally active complex, β-catenin must recruit one of the two Kat3 transcriptional coactivators, Kat3A, cAMP response element binding protein [CREB-binding protein (CBP)] or its closely related homolog Kat3B, p300 (E1A-binding protein, 300 KDa)^[43,97]^ to promoters and enhancers. Kat3 coactivators, by binding to hundreds of proteins, play critical roles as master regulators of transcription. Kat3 activation has been previously reviewed by our team^[23,41]^, and is driven by multiple signals including Wnts, high glucose, hypoxia, and EMT inducers. Historically, CBP and p300 have been considered largely redundant due to their significant protein sequence identity and even higher similarity. However, CBP and p300 are clearly not redundant and carry out definitive and unique roles both *in vitro* and *in vivo*^[23,98–101]^. From a library of 5000 secondary structure mimetics, we identified ICG-001 (IC_50_ = 3 μM) in a Wnt reporter screen in colon cancer cells. We subsequently identified and validated that the molecular target of ICG-001 was CBP and that ICG-001 binds specifically and with high affinity (_~_1 nM) to the N-terminus of CBP but not to p300^[102,103]^. We subsequently found that selectively blocking the CBP/catenin interaction with ICG-001, with an increase in p300/catenin-mediated transcription leads to the initiation of differentiation in stem and progenitor cells including ES, iPS, SSC and CSC [Figure 3A]^[104–108]^. These investigations allowed us to propose our model of differential coactivator usage. The critical non-redundant roles that CBP and p300 play in catenin-mediated transcription are highlighted in our model ^[109]^ [Figure 3B]. The model posits that catenin’s choice to utilize either CBP or p300 is the first decision that guides a stem cell to either maintain potency or initiate a differentiative transcriptional program, respectively [Figure 3B]. We subsequently identified several small molecules, IQ-1 and ID-8, which are indirect p300/catenin inhibitors as well as the specific direct p300/catenin antagonists YH249/250. P300/catenin antagonists maintain the potency (pluri-or multipotency) of both mouse and human embryonic, induced pluripotent and somatic stem cells, by increasing CBP/catenin driven symmetric divisions both *in vitro* and *in vivo*^[107,109–112]^ [Figure 3C].

**Figure 4.**
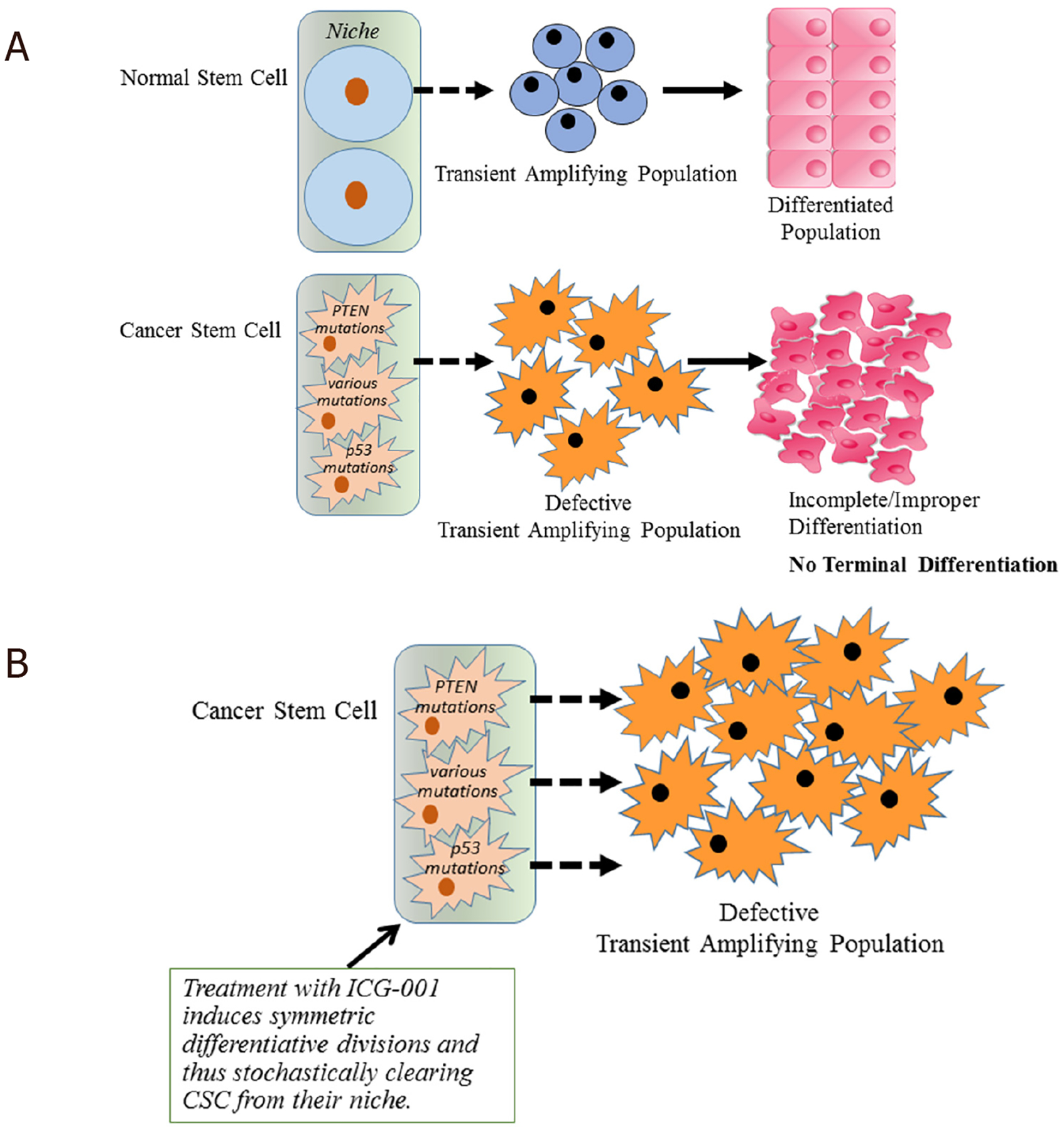
Intrinsic differences in the mode of division of SSC and CSC allow for the safe elimination of CSC via symmetric differentiative divisions. A: Asymmetric division is preferred in normal somatic stem cells (SSCs). Both symmetric and asymmetric divisions occur in cancer stem cells (CSC), thereby leading to an increase in the CSC population; B: CBP/catenin antagonists (e.g., ICG-001) force symmetric differentiative divisions in CSC thereby driving the CSC population out of their niche. CBP/catenin antagonists maintain SSC asymmetric divisions thereby never depleting the niche

We have extensively examined the therapeutic potential of selectively antagonizing the CBP/catenin interaction, and have demonstrated the ability to safely eliminate drug-resistant CSC, via forced differentiation, without deleterious effects on the normal endogenous stem cell populations^[104,105,113–115]^. CBP/catenin antagonists can activate SSC and induce asymmetric differentiation thereby enhancing repair pathways in preclinical models of pulmonary and renal fibrosis^[116,117]^, myocardial infarction^[118]^ and neurodegeneration^[23,108,119]^. The differential effects of CBP/catenin antagonists on CSC versus SSC, specifically forced differentiation and elimination versus differentiation and enhanced repair without depletion, are cell intrinsic. CBP/catenin antagonists utilize the intrinsic propensity of CSC to preferentially divide symmetrically^[36,37]^ thereby stochastically eliminating CSC via forced symmetric divisions [Figure 4A]. SSC preferentially differentiate asymmetrically, with one daughter cell always remaining in the niche and therefore are not depleted [Figure 4A]^[50]^. Asymmetric differentiation can be activated by CBP/catenin antagonists thereby enhancing repair without damaging the normal SSC population^[23]^. Therefore, CSC when treated with CBP/catenin antagonists will stochastically be cleared from their niche via symmetric differentiative divisions [Figure 4B].

Significant concerns about specificity arise when targeting the coactivator protein CBP, as it has as many as 500 molecular partners, including a vast array of transcription factors^[119]^. It is important to note that neither pre-clinical nor clinical studies have shown toxicity when utilizing specific small molecule CBP/catenin antagonists are safe. PRI-724 (IC_50_ ~150 nM), a second-generation clinical CBP/catenin antagonist demonstrated an excellent safety profile in preclinical IND enabling toxicology studies. The no-adverse-event-level for PRI-724 in dogs was 120 mg/kg/day administered for 28-day via continuous infusion^[120]^. Clinically, PRI-724 had an excellent safety profile, demonstrating no dose limiting toxicities with escalation from 40 to 1280 mg/m^2^/day administered by continuous i.v. infusion. Down regulation of the biomarker *survivin/BIRC5* with upregulation of the differentiation antigen *CK20* in EpCAM selected circulating tumor cells strongly correlated with increasing plasma concentrations of drug^[120]^. PRI-724 also demonstrated safety and efficacy with increased liver function in a trial conducted in patients with HCV-induced hepatic fibrosis^[121]^.This degree of safety was initially surprising. We believe this is due to the high biochemical specificity of ICG-001/PRI-724 for binding to CBP, and its limited impact on only a fraction of all CBP interactions. The unique non-redundant roles that the N-termini of the two Kat3 coactivators CBP and p300 play in stem cell biology and the intrinsic preference for asymmetric division in normal SSC are critical to the safety of these agents.

### Kat3A/CBP and Kat3B/p300 and SSC Metabolism

“Nothing in Biology Makes Sense Except in the Light of Evolution” - Theodosius Dobzhansky.

**Figure 5.**
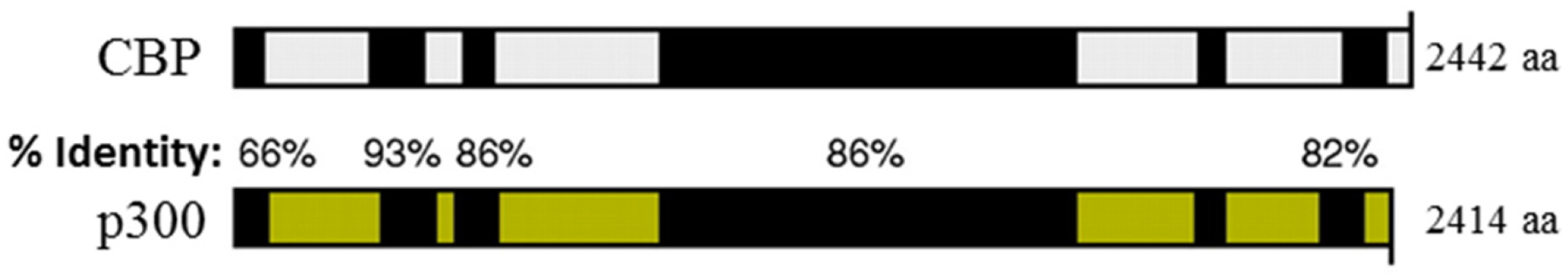
Despite having diverged more than 450 million years ago, CBP and p300 possess a very high percentage of identity and even higher homology at the amino acid level. The most divergent region by far is the very amino termini of CBP and p300 to which ICG-001/PRI-724 and YH249/250 bind respectively

Quiescence provides safeguards the functionality of SSC by restricting the damage caused by mitochondrial respiration and reactive oxygen species generated during oxidative phosphorylation. These safeguards limit DNA mutations and prevent uncontrolled cell cycle entry^[122,123]^. SSC and CSC preferentially utilize glycolysis over oxidative phosphorylation despite the inefficiency in regards to ATP generation of glycolysis compared to oxidative phosphorylation^[124]^. The activation of quiescent SSC and the initiation of differentiation involves a metabolic change from glycolysis and entry into the Krebs cycle. Reprogramming to pluripotency, on the other hand is associated with “anaerobicizing”^[125]^. With the dawn of the evolution of vertebrates, roughly 450 million years ago, a new lifestyle having a relatively long-lived adult stage began. To accommodate this successfully a mechanism for long term homeostatic maintenance and tissue repair was essential. This was accomplished via quiescent “immortal” SSC maintaining an “anaerobic” metabolic state in specialized niches as opposed to their more proliferative aerobic-differentiated daughter cells. This mechanism evolved in order to protect genetic material integrity in long lived vertebrates^[126]^. Maintaining the two different populations resulting from asymmetric division; one daughter being a long-lived quiescent SSC utilizing anaerobic metabolism and the other a rapidly expanding differentiating population utilizing aerobic metabolism, required tight regulation. The Kat3 coactivator family CBP and p300 diverged via gene duplication just prior to the vertebrate radiation over 450 million years ago^[127]^. CBP and p300 are extremely large proteins encoded over 33 and 31 exons respectively. CBP and p300 retain extremely high identity, up to 93%, particularly over a large central core that includes the CH1, KIX, Bromodomain, and CH2 and CH3 regions [Figure 5], despite diverging over 450 million years ago^[128,129]^.

The small molecules CBP/catenin antagonists, ICG-001/PRI-724, and p300/catenin antagonists, YH 249/250, bind the CBP and p300 N-termini, respectively^[23,102,111,130]^. This least conserved region between the two coactivators, which has only 66% identity, binds both β-catenin and through a highly conserved LXXLL sequence, nuclear receptor family members^[131]^. The N-termini within each orthologous coactivator are extremely conserved with human and mouse CBP being 98% identical at the amino acid level within this region. The nuclear receptor family and Wnt signaling appeared significantly earlier in evolution approximately 600 million years ago in the first multicellular animals (metazoans)^[132,133]^ and are found in nematodes, flies, and vertebrates.

Previously, we proposed that gene duplication generated the two Kat3 coactivators and a subsequent rapid divergence within their N-terminal regions occurred at the same time as the integration of Wnt and nuclear receptor family signaling^[23]^. This co-evolution resulted in high fidelity control over the differential cell fates generated by asymmetric stem cell division, thereby enabling two inherently different cell populations and providing the expanding daughter cell population integrated pathways to generate divergent cell types. This joint genetic divergence and signaling integration additionally provided a mechanism to “read” multiple signals affecting SSC quiescence and DNA integrity. For example, a huge number of lesions in DNA can be induced by ultraviolet light^[134]^.Therefore, circadian regulation of normal SSC activation and division is critical^[135]^. Shift workers, with aberrant circadian regulation, have an increased risk for the development of cancer^[136,137]^. Metabolic and circadian regulation control the timing and the mode of SSC division^[138]^ and metabolic pathways through nuclear receptors (e.g., PPAR, Rev-Erbα and β) play critical roles in circadian integration of metabolism energetics^[139,140]^. Clock, part of the master circadian regulatory circuit mediated by the Clock/Bmal1 transcriptional complex, is recruited to p300 *in vivo* in a time-dependent manner^[141]^. Evolutionarily, it would seem logical that mechanisms to enhance SSC asymmetric differentiation and symmetric differentiative divisions of CSC or pre-CSC would have evolved. In fact, numerous naturally occurring CBP/catenin antagonists have evolved. Returning to the concept of differentiation therapy, all-trans retinoic acid (ATRA), a vitamin A derivative, via its nuclear receptor complex (RAR/RXR) acting as a CBP/catenin antagonist is very effective in treating Acute Promyelocytic Leukemia. ATRA, similar to ICG-001 does not kill malignant cells but rather induces them to differentiate. Vitamin D plays an important role in cancer prevention through the (VDR/RXR) nuclear receptor complex and both ATRA and VitD have been shown to antagonize aberrant Wnt signaling in the context of malignancy^[142]^. Nuclear receptor family members, via competition with β-catenin for binding to the N-terminus of CBP, phenocopy CBP/catenin antagonists. However, synergistic effects on the activation of gene expression by nuclear receptors and Wnt signaling have been demonstrated (e.g., ATRA and Wnt)^[143]^ and nuclear receptors also on their own control the expression of various transcriptional cassettes. Thus, nuclear receptor family members are not simply “pure antagonists” of CBP/catenin transcription and therefore have significant differences from small molecule direct CBP/catenin antagonists.

The LXXLL sequence present in the amino termini of both CBP and p300 is highly conserved and can recruit both RAR/RXR and VDR/RXR complexes, and potentially all other nuclear receptor complexes including AR, PPAR, and others. Not surprisingly, multiple nuclear receptors can effect stem cell maintenance or initiate differentiation in a manner similar to small molecule p300/catenin or CBP/catenin antagonists^[23,144]^. However, in contrast to modulation of nuclear receptors, which can cause developmental defects, selectively antagonizing the CBP/catenin interaction with ICG-001, even at very high levels, is extremely safe and has no deleterious effects on mouse embryonic development^[118,145]^. Female mice treated topically or orally with high doses of ICG-001 throughout pregnancy have normal litters. The pups exhibited normal weight and size compared to their control littermates and can reproduce normally, demonstrating no deleterious effects to germ cell populations, which interestingly, also prefer asymmetric divisions^[146,147]^. Interestingly, a 27 bp/9aa deletion in CBP between the β-catenin-binding region (DELI-sequence) and the nuclear receptor (LXXLL) binding sequence is a strongly evolutionarily conserved. Using CRISPR/Cas9 editing of p300, we recently demonstrated that this deletion in CBP provided a mechanism via steric inhibition, for nuclear receptors to antagonize CBP/catenin signaling, allowing for the maintenance of quiescence and initiation of asymmetric divisions in SSC. Whereas β-catenin and nuclear receptor signaling can synergize to effect a feed-forward mechanism to drive differentiation and lineage commitment utilizing p300, as steric constraints removed by the conserved 9 amino acid insertion is sufficient to allow for the simultaneous binding of nuclear receptors and β-catenin^[130]^.

### Summary: CSC resistance and differential Kat3 coactivator usage

SSC and CSC utilize Wnt/catenin signaling and differential Kat3 coactivator usage to regulate stem cell homeostasis and the balance between self-renewal and differentiation. The fundamental difference between SSC and CSC appears to be a preference for asymmetric over symmetric divisions respectively. Increased CBP/catenin transcription is associated with enhanced telomerase activity and the expression of *BIRC5/Survivin*^[98]^ required for self-renewal of stem cells. In this regard, targeting CBP/catenin signaling appears to represent a common “Achilles’ Heel” in CSC in both solid and liquid tumors^[23,104,113,148]^. Aberrant regulation of catenin/Kat3 coactivator usage enhances CBP/catenin activation at the expense of p300/catenin-mediated transcription. Preferential use of this coactivator can arise from a vast array of mutations, either inherited or acquired, and a wide variety of insults (i.e., chronic inflammation, viral infection, high fat/caloric diet, and others). Resistance to therapy, radiation, chemotherapy of immunotherapy is associated with selection of resistant clone(s) from a pre-existing CSC pool. CBP/catenin antagonists, by taking advantage of this fundamental difference between SSC and CSC can safely stochastically differentiate away symmetrically dividing CSC without depleting the SSC population that is dividing symmetrically. However, in cancer, the transient amplifying population is not sensitive to CBP/catenin antagonists and still must be targeted to eliminate the disease, as these populations rely on other pathways (Bcr-Abl, KRAS, etc.) to maintain their non-terminally differentiated proliferative status^[104,105,114]^. The robust safety profile of CBP/catenin antagonists could eventually provide an opportunity to utilize them in a “vitamin-like” manner as a prophylaxis to the accumulation of pre-CSC or CSCs.

## Figures and Tables

**Table 1:** Wnt Target Genes Associated with “Stemness”

Genes	References
Stemness related	
Survivin/BIRC5	[58]
htert	[59]
Cdx1	[66]
Id2	[67]
LGR5/GPR49	[68]
CD44	[69]
CD24	[70]
CD133	[71]
ABC cassette genes	[72,73]
EpCAM	[74]
EMT related	
Slug	[60]
S100A4	[62]
Fibronectin	[62]
L1CAM	[62]
CD44	[62]
MMP7	[62]
uPAR	[62]
